# Electronic Cigarette Marketing Online: a Multi-Site, Multi-Product Comparison

**DOI:** 10.2196/publichealth.4777

**Published:** 2015-09-11

**Authors:** Kar-Hai Chu, Anupreet K Sidhu, Thomas W Valente

**Affiliations:** ^1^ University of Southern California Department of Preventive Medicine Los Angeles, CA United States

**Keywords:** electronic cigarettes, content analysis, social networking sites, marketing

## Abstract

**Background:**

Electronic cigarette awareness and use has been increasing rapidly. E-cigarette brands have utilized social networking sites to promote their products, as the growth of the e-cigarette industry has paralleled that of Web 2.0. These online platforms are cost-effective and have unique technological features and user demographics that can be attractive for selective marketing. The popularity of multiple sites also poses a risk of exposure to social networks where e-cigarette brands might not have a presence.

**Objective:**

To examine the marketing strategies of leading e-cigarette brands on multiple social networking sites, and to identify how affordances of the digital media are used to their advantage. Secondary analyses include determining if any brands are benefitting from site demographics, and exploring cross-site diffusion of marketing content through multi-site users.

**Methods:**

We collected data from two e-cigarette brands from four social networking sites over approximately 2.5 years. Content analysis is used to search for themes, population targeting, marketing strategies, and cross-site spread of messages.

**Results:**

Twitter appeared to be the most frequently used social networking site for interacting directly with product users. Facebook supported informational broadcasts, such as announcements regarding political legislation. E-cigarette brands also differed in their approaches to their users, from informal conversations to direct product marketing.

**Conclusions:**

E-cigarette makers use different strategies to market their product and engage their users. There was no evidence of direct targeting of vulnerable populations, but the affordances of the different sites are exploited to best broadcast context-specific messages. We developed a viable method to study cross-site diffusion, although additional refinement is needed to account for how different types of digital media are used.

## Introduction

Electronic cigarettes, also known as e-cigs or e-cigarettes, are battery-operated products that deliver nicotine by turning it into an aerosol that is inhaled by the user [1]. Today, awareness of e-cigarettes is high [2], and their use continues to grow [3,4]. With declining cigarette sales and the potential for increasing Food and Drug Administration (FDA) regulation, there is a rise in noncombustible tobacco products, with e-cigarette advertising being the most extensively circulated [5]. E-cigarette brands have been using widespread advertising campaigns, spending $541.7 million in 2011 [6].

The Internet represents a medium that offers an opportunity for e-cigarette brands to expand their audience reach. However, this unregulated domain can also be a cause for concern. Several studies have suggested that e-cigarettes can be a viable method to quit smoking [7,8], although Grana and Ling [9] found that health claims and smoking-cessation messages on many websites, aided by images of doctors or celebrities, are unsupported by scientific evidence. Misinformation can be easily spread in many arenas online, and social networking provides a convenient and cost-effective venue for e-cigarette promotions of non-scientifically supported claims.

There are countless social networking sites with unique technological features and user demographics that can be attractive for selective marketing. The growth of e-cigarettes occurred as social media and other Web 2.0 sites became an important platform for commercial advertising. It is no surprise that e-cigarette brands, especially smaller ones that have no affiliation with larger tobacco companies and no sizable advertising budgets, took to social media to market their products. Recent e-cigarette studies have typically examined product health effects (eg, [10,11]). Few studies, however, have examined e-cigarette marketing within social media. In one example, Huang et al [12] found that e-cigarette tweets were mostly commercial (90%), and were posted by a small group of active accounts. They concluded that Twitter served as an important platform for e-cigarette marketing.

Social networking sites such as Facebook or Twitter are inherently built to support social networks. Whether posting messages to a Facebook wall or sending updates on Twitter, interactions between users are the foundation of all activity on these sites. The distinction in how users of different sites interact depends on the digital media affordances the sites focus on. Affordances are the actionable relationships between an actor and an aspect of the environment that offer the actor the potential for action [13]. This concept has been extended to human-computer interactions and applied to product designs, such as graphical user interfaces [14]. The concept of affordances is especially important as new technologies continue to be developed.

Web 2.0 tools provide an abundance of ways to interact, and social networking sites use these tools to great advantage. Kietzmann and colleagues’ well-cited work [15] describes seven functional blocks of social networking sites: identity, conversations, sharing (of content), presence, relationships, reputation, and groups. Each site varies in the degree to which they use or promote these functions. For example, every Instagram post has a picture, and sharing is at the core of the site. Twitter, on the other hand, allows users to micro-blog by producing 140 character tweets that promote sharing and conversations. Facebook and Google+ combine various digital media, wrapped together to create a general social networking platform, focusing on relationships and identity. Indeed, Facebook friends are not the same as Instagram friends, and spreading messages on Twitter is different than doing the same on Google+. These differences in digital media afford unique ways by which users on these sites interact, and can result in different kinds of relationships being developed [16].

Social networking sites also have varying user demographics. A Pew 2013 report on social media [17] revealed that Facebook has an increasingly older population (users 65+), Twitter has high adoption by African Americans, women are 4 times more likely than men to be Pinterest users, Instagram is also popular among women, and LinkedIn is popular for higher-income households. Research using different methods of collecting demographic data, such as connecting self-reported user names with ethnicity based on US Census data, report similar results for Twitter [18] and Facebook [19]. There are other demographic trends, but it is clear that each social networking site has different levels of usage and appeal for certain demographics. These variations in social networking site demographics allow marketing companies to customize their advertisements and possibly target sub-populations based on their social networking tendencies.

The unique affordances and demographics of each social networking site, especially how they are used by online marketers, is the focus of this paper. In this study, we explore the possibility of e-cigarette brands potentially taking advantage of social networking site demographics and targeting vulnerable sub-populations, such as underage youth. We will also see if and how e-cigarette brands utilize different digital media in their marketing strategies. As such, we ask two primary research questions: (1) Are e-cigarette brands exploiting the affordances of each site in their marketing? And (2) Are e-cigarette brands targeting sub-populations (eg, women, teenagers) by taking advantage of the demographic differences of different social networking sites?

We also study how different social networking sites potentially interact with one another, for example when posting a tweet to one’s Facebook wall. To our knowledge, no studies have examined the diffusion of information across multiple social networking sites. However, Pew’s latest (2014) survey indicates that 52% of online adults use two or more social media sites, rising from 42% in 2013 [20]. Given our plans to collect information across multiple social networking sites, we are also interested in determining if viable methods can be developed to identify how users and information might cross the boundaries between different platforms. There are many implications in this line of study, including diffusion of messages, how different technologies can change message content, and roles that people might serve in bridging social networks from different sites, to name a few. This leads to our final exploratory research question: Are there identifiable instances of users and information crossing different social networking sites?

## Methods

### Data

This study examined four social networking sites and two e-cigarette brands that maintain a presence in each site for marketing and advertising. Data from each of the four sites were collected from October 20, 2012 through April 14, 2015. The sites were chosen based on popularity (from current eBizMBA.com rankings), differences in the affordances of the platform’s technologies, accessibility of data, and user demographics [20,21]. The final set of sites included Facebook (general purpose, with increase usage among seniors), Twitter (text micro-blog, with high adoption by African Americans), Google+ (general purpose), and Instagram (picture based, with high adoption by women). E-cigarette brands were chosen based on activity in multiple social networking sites and general online presence. The two choices were Blu (owned by Lorillard, then by Reynolds Tobacco, and finally by Imperial Tobacco in 2014) and V2 (owned by VMR). Currently, Blu and V2 are two of the top three e-cigarette brand websites based on activity, according to traffic tracker Compete.com, with similarly high social media activity. Blu also has the highest advertising expenditures, comprising more than 75% of all e-cigarette advertising expenditures in 2012 [22]. These two choices also allow us to see potential differences between a tobacco industry-owned brand (Blu) and a privately owned brand (V2).

Data were collected by establishing connections to each social networking site via the platform’s Application Programming Interface (API). An API allows external software to make requests for data and post information and to perform tasks that are made allowable by the service. Custom software was written to communicate with the API of each of the four social networking sites. The 3 main objectives were to:

1. Gather any available long-term historical data, primarily any content posted by Blu or V2 and related information, such as users who shared or commented on such posts. This process was conducted a single time.
2. Gather recent data as it was being posted. This task was the same as objective 1 but only collected recent data (about 3-4 days old), as many social networking sites limit the amount of data that can be requested. This process was executed every 3-4 days.
3. Gather related data unique to each site, as their methods of online interactions are different. For example, the data collection included retweets on Twitter, comments on Facebook, shares on Google+, likes on Instagram, and so on. Users who performed these actions were also recorded, along with any available demographic information. This process was executed every 3-4 days, in parallel to objective 2.

All data collected were publicly available since any person with an Internet connection is able to view data that has been retrieved through this mining software. The primary data being collected were content posted on the social networking sites. Demographic information came from users’ self-reported data in the site and was only collected if the user had made it public. Personal information such as emails, phone numbers, or addresses was not collected. This study was reviewed by the University of Southern California Health Sciences Institutional Review Board, which found that it does not qualify as Human Subjects Research and thus is not subject to the requirements of 45 CFR 46.102.

### Analysis

We analyzed the content posted on each of the social networking sites and parsed data according to e-cigarette brand and social networking site. Our primary method of analysis was Term Frequency-Inverse Document Frequency (TF-IDF) weighting a commonly used statistical method in information retrieval to calculate word relevance for a document across a large corpus [23]. TF-IDF classifies documents by examining each word and calculating its occurrence frequency. TF is the measure of importance of a single term in one document, as determined by its frequency (usually normalized by the total number of words in the document). The TF score is balanced by the IDF, which counts the number of documents a term appears in across the entire corpus, and takes the inverse. Thus, IDF measures the discriminating power of a term. In short, TF-IDF classifies any single document by terms that frequently appear in it but do not appear in many other documents. More advanced techniques can also build from this foundation, although they were not used in this study. We used the Simstat 2.6.2 software package and its associated content analysis component, WordStat 6.1.23, to support the analyses.

We conducted an exploratory network analysis of users who had posted content or interacted with one of the e-cigarette brands in one of the four social networking sites. To examine the possibility of cross-platform diffusion of e-cigarette messages, we ran the test in two steps. First, we identified usernames from each site that had an identical matching username in another site. While this tactic did not guarantee certainty that the different users are the same, it provided a starting approximation. We then analyzed content posted by the identified users in the different sites. Using the names that were found in multiple sites, we confirmed it was in fact the same person via identical profile pictures or personal descriptions, and then examined whether their activity on one site (eg, liking a Blu post on Facebook) resulted in sharing the content on another site (eg, posting the same message on Twitter).

## Results

Table 1 describes the data, including total counts of posted content on each of the four social networking sites. Table 2 is a list of the top 20 terms used based on TF-IDF score. These are the top terms based on frequency and number of cases they appear in (representing how unique they are for each possible brand/site combination). The terms help contextualize the cases. As we are studying four social networking sites and two e-cigarette brands, there are 8 possible brand/site scenarios, or cases, that any given term can appear in. For example, the term *ecig* is used by both companies in all four sites, and thus has a count of 8 for number of cases, also called an 8-case scenario. In another example, the term *RT* is used by both companies but only on Twitter, and thus has a count of 2 for number of cases.

**Table 1:** Summary of posted data collected.

	Facebook	%	Google+	%	Instagram	%	Twitter	%
Total	9915		371		638		7861	
Blu	6313 (2106) ^a^	64% (61%)	335	90%	342	54%	4753	60%
v2	3602 (1343) ^a^	36% (39%)	36	10%	296	46%	3108	40%

^a^Facebook data includes posts by other users to be displayed on the e-cigarette brand page. Number in parenthesis represents posts made by the e-cigarette brands, ie, Blu and V2.

**Table 2:** Top terms based on TF-IDF scores.

	Frequency	% Total	No. cases	% cases	TF-IDF
RT	1200	0.50%	2	25.00%	722.5
BLUCIGS	1827	0.80%	4	50.00%	550
CO	4111	1.80%	6	75.00%	513.6
HREF	559	0.20%	2	25.00%	336.6
BLU	1302	0.60%	5	62.50%	265.8
NOFOLLOW	409	0.20%	2	25.00%	246.2
REL	409	0.20%	2	25.00%	246.2
BLUCRM	272	0.10%	1	12.50%	245.6
DM	325	0.10%	2	25.00%	195.7
OT	410	0.20%	3	37.50%	174.6
BLUNATION	556	0.20%	4	50.00%	167.4
CLASS	466	0.20%	4	50.00%	140.3
SAVE	503	0.20%	5	62.50%	102.7
PWD	110	0.00%	1	12.50%	99.3
WARD	163	0.10%	2	25.00%	98.1
VAPORIZER	206	0.10%	3	37.50%	87.7
SXSW	202	0.10%	3	37.50%	86
HASHTAG	412	0.20%	5	62.50%	84.1
BLUFREEDOM	275	0.10%	4	50.00%	82.8
HTTPS	589	0.30%	6	75.00%	73.6

In contrast, a 1-case scenario shows words that are only found in a single brand/site case (eg, *BLUCRM* or *PWD* in Table 2). In other words, those terms were only used by a single company on a single site. These terms are helpful in explaining why only one brand might be applying a specific marketing strategy on a single site. Applied to only a single case out of eight possibilities, it is the most conservative classification of the case by the terms found. These terms are unique, based on either the brand’s usage or how the technology supports certain features. We found 163 terms that fit the 1-case scenario and classified each according to 15 possible categories. The categories were developed through an exploratory examination of the data, and the terms were coded by two of the authors. The authors agreed on the categories of 121 of the terms (74%). The remaining disagreements in classifications were discussed until a mutual agreement had been reached for all 163 terms. The most frequent categories found in the data were: 68% of Blu’s terms on Twitter were for user interactions; 84% of Blu’s terms on Facebook were for political information; and 73% of V2’s terms on Twitter were links to their homepage. No strong content data were found to help classify either brand’s activities on Google+ or Instagram, or for V2 on Facebook.

The results showed that Blu and V2 had contrasting strategies in their social networking site presence. In the 1-case data, V2 focused primarily on Twitter (94% of all of V2’s 1-case terms), with the majority of interactions aimed at connecting followers to their home website. V2 did not have any notable 1-case discriminating content on Facebook, Google+, or Instagram. On Twitter, V2’s content remained focused on website advertisement, although interactions with users were also included. Blu had unique content in both Twitter (66%) and Facebook (28%). Unlike V2, Blu’s use of Twitter focused on interacting with users on a wide range of topics, from product support to general conversation. On Facebook, Blu’s posts centered on political activities (eg, suggestions to email state representatives or city council members, information on rallies, etc). Blu had no significant unique activity on Google+ or Instagram. Here are content examples from several of the top categories:

(Blu/Twitter/User interaction) @anonymized_username but that doesn't say, "I'm either really embarrassed or really proud to wear this at Christmas".

(Blu/Twitter/Event info) We've seriously had Fun Fun Fun giving out these @funfunfunfest tix #FFFfest

(Blu/Facebook/Political info) Did you see where a local Wisconsin legislature wants to pass a bill ensuring e-cigs are allowed to be used in public? Hit LIKE if you support this!

(V2/Twitter/Website) If you want to try some NEW flavors on your V2 battery, check out our clearance section!

(V2/Twitter/User interaction) @anonymized_username Nice V2 stash! Happy vaping! :)

A second follow up was conducted to investigate the terms in each brand/site with the highest raw frequency by removing any discriminating factor based on IDF. In addition, we filtered out site-specific terms (eg, *RT* in Twitter or *href* in Google+) to focus on terms that are topic-specific (ie, focused on e-cigarettes). Without the IDF discriminator or site-specific terms, these data provide a broad view of all general-purpose terms that are used in each site, according to each brand. We were able to see what themes and concepts the e-cigarette brands were broadcasting to their followers, regardless of which site they were using. The results are shown in Table 3.

Table 4 is a matrix showing the number of users that share a presence between two given sites. The diagonal shows the total number of users that had activity within a single site. A small percentage of matched screen names were found between profiles of users in the different sites. In terms of raw frequency, Instagram/Twitter had the most matches, followed by Instagram/Facebook, and Twitter/Facebook. We selected the top three users found in each site case, with six possible cases (three pairs of two), for 18 potential users. All users were confirmed to be the same across sites by either identical images or profile descriptions. In each of the 18 cases, we found that no identical content crossed site boundaries.

Table 5 shows non-content interactions between users and an e-cigarette brand, separated into three types: 1) *comments*, which include text responses to posted content, 2) *likes/plusoners*, which includes a single supporting action that is collectively aggregated, and 3) *resharers/retweets*, which are actions where users repost existing content. These actions were not fully inclusive, as we only collected those that were relevant to this study.

**Table 3:** Top terms by raw count, with website coding terms removed.

Blu				V2			
Facebook	Google+	Instagram	Twitter	Facebook	Google+	Instagram	Twitter
Blu	vaping	blucigs	blucigs	cigs	products	cigs	cigs
blucigs	blunation	blunation	blu	shop	cig	vapor	new
cigarettes	blucigs	vaping	blucrm	new	ecigs	vaporizer	flavor
Ward	blufreedom	blufreedom	thanks	day	vapor	ecig	ecig
New	blu	blu	customer	save	liquid	vaping	save
Ecigs	rewards	vapelife	blunation	flavor	blog	ecigs	liquid
electronic	freedom	vaporlounge	call	products	categories	vape	vaping
Day	ecigs	indycar	sxsw	sale	shirt	vapesess	kit
Cigs	rewards	electriclounge	new	cig	standard	flavor	sale
Org	freedom	daysofblu	help	kit	mens	sale	day

**Table 4:** Cross-site users.

	Facebook	Twitter	Instagram	Google+
Facebook	18504	32	60	0
Twitter	--	2048	128	0
Instagram	--	--	3613	0
Google+	--	--	--	266

**Table 5:** Non-content interactions between users and e-cigarette brands. The numbers in parentheses represent the ratio of comments to original posts on Facebook and Instagram.

Facebook	Count	Google+	Count	Instagram	Count	Twitter	Count
comments	26612			comments	2127		
Blu	18582 (2.94)			Blu	1838 (5.37)		
V2	8030 (2.23)			V2	289 (0.98)		
Likes	54029	plusoners	754	likes	17034		
Blu	37231	Blu	740	Blu	11214		
V2	16798	v2	14	V2	5820		
		resharers	80			retweets	14781
		Blu	80			Blu	13879
		V2	0			V2	902

## Discussion

### Exploring the Results

The 1-case data provides valuable information in determining some of the most discriminating terms used by each e-cigarette brand, for the different social networking sites. It also reveals early evidence of how the two e-cigarette brands differ in their social media marketing strategies, with V2 focusing on marketing products on their website and Blu using Twitter for user interactions and Facebook for political activity information. The 163 terms were able to show that specific combinations, specifically Blu/Twitter, Blu/Facebook, and V2/Twitter, were being utilized by each brand for a particular type of marketing. However, the limited data—only 163 terms—does not provide enough information for deeper investigations. Therefore, we viewed the data through several other lenses. First, we examined the n-case data to include all possible terms (Table 2). In many of these cases, there were technologically driven explanations for the high discriminating power of some of the terms. For example, *RT* has the highest TF-IDF score and is found in only two case scenarios (Blu/Twitter and V2/Twitter). However, this is not unexpected, as it is only used in Twitter as shorthand for “retweet”. Similarly, terms such as *href* or *nofollow* are webpage-coding syntax used only in Google+ to create links to external sites, usually to images or videos. Interestingly, Instagram is the only site where coding terms are not used, another artifact of the available technology. As an image-based platform, there would be no need for text to contain any additional image links for each Instagram post. Other types of links seen in Facebook or Google+ were not seen in Instagram captions. Overall, the n-case results suggest that properties inherent to specific platforms can dictate the types of discriminating content found.

We continued to broaden our view by removing the discriminating IDF and also filtering out technology-specific terms (Table 3). These results help provide additional evidence of the marketing strategies of each e-cigarette brand on the different sites. When looking at the terms for V2, we found a similar theme as in the 1-case results: V2’s focus is on brand marketing, different products, and directing users to their website. In the case of Blu, we again confirmed some of the findings from the 1-case view. Terms such as *thanks*, *customer*, and *help* in Twitter were indicative of interactions with their followers. The only political term, *ward* (in context of geopolitical boundaries), was found only in Facebook. Interestingly, we saw a theme across Blu’s social networking site presence, containing the terms *blunation* and *blufreedom* in Google+, Instagram, and Twitter. Blu’s social media strategy appears to focus more on community and lifestyle, contrasting sharply with V2’s efforts to market products and direct users to their website. Blu’s efforts are more engaging than V2, possibly leading to more conversations and additional activities across the sites. Table 5 shows evidence of user engagement; user comments represent actions when users are responding in conversation to an original post, as compared to likes or redirects, which typically only require a single mouse click. In both sites where comments were recorded (Facebook and Instagram), Blu followers commented at higher rates than V2.

The cross-platform network analysis found several meaningful results (Table 4). First, the users on Google+ appeared to share no connection with any of the other sites. One possible reason for this is that Google+ does not explicitly require a traditional screen name that serves as an alias, but instead concatenates what the user inputs for a first and last name. Because extra steps are needed to create a pseudonym, users are more likely to retain Google+’s universal naming scheme, resulting in different aliases used than on other sites. Second, the highest number of overlapping screen names was between Instagram and Twitter. We had expected Facebook to be one of the overlapping pairs based on the high number of collected Facebook users and Pew’s survey of users with multiple social media accounts [20], leading to a much greater potential for matches. However, as Instagram and Twitter have a more distinct focus on their media usage—short text for Twitter, images for Instagram—it is possible that users would overlap in their usage of these two sites rather than the more general social networking tools of Facebook. Overall, we expect that that our results are likely to represent the minimum amount of overlapping users, as it is likely that users might maintain different online identities and change their screen names in different sites.

The content analysis of cross-platform posts yielded no results where activity from a user on one site led to sharing that message on a different site. While our methods were sound, we found several limitations to consider. When we searched for identical content between Instagram and another site, either Twitter or Facebook, we found that an image-based post would be difficult to forward to another site; the user would need to save the image and repost it, otherwise the caption text would not make sense out of context. Conversely, a message on Twitter without an image would not be posted on Instagram. Also, it is important to consider that the nature of one’s network of friends on each site might be distinct. Different sites might be used to develop and maintain different relationships [16]. People might be using Facebook to connect with close family and friends who may not be supportive of smoking/e-cigarette use, therefore making Facebook an unbefitting platform to post about e-cigarettes. Twitter, on the other hand, allows users to connect directly with the staff and management of e-cigarette companies, which makes it easier for them to communicate and seek support directly through tweets. This is also apparent from the increased focus of the companies on Twitter use (V2=94%, Blu=66%). Another limitation was in attempting to study any content posted on Facebook, as most users had made their accounts private.

While not directly related to our research questions, we were also able to observe some of the consequences of each brand’s marketing strategy. We compared the available interactions that users could have with each e-cigarette brand for the four websites; these included Facebook’s comments and likes, Google+’s plusoners and resharers, Instagram’s comments and likes, and Twitter’s retweets. In every case, Blu’s followers always had higher percentages of interactions (see Table 5). This suggests that Blu’s efforts at interacting with their user base, rather than V2’s strategy of directing traffic to their website, is more successful at engaging users, eliciting responses, and raising interest. These strategies might be based on the differences between an independent brand and one owned by a tobacco company. Additional studies with other brands will be necessary to determine if parent-company ownership has any effect.

### Addressing the Research Questions

In addressing the first research question—Are e-cigarette brands exploiting the affordances of each site in their marketing?—we found evidence that Blu and V2 were using the sites in different ways, likely utilizing the affordances made available. The methods by which each brand interacted with its audience, advertised products, or relayed information aligned with the affordances of each platform. We could see Blu’s usage of Twitter as a medium to interact with its followers, frequently mentioning users by name, and directly conversing with them. Twitter’s @user_mention function, and an interface that allows users to immediately see Blu’s interactions with them, all support an interactive environment. Facebook’s “wall” feature offers a different format, in which users immediately see all other posts connected to a single parent discussion message. This affords a system whereby mass broadcasting is effective, reflected in the high number of political announcements and news events posted, with almost no direct user interaction. Kietzmann et al’s [15] view of Facebook’s affordances reflects this, as they note the importance of relationships over conversations. Twitter, on the other hand, prioritizes conversations over relationships, supporting Blu’s usage. V2 similarly utilized Twitter functions for easy interaction with individual users, although they consistently used all of the sites as a way to link users back to their homepage.

In addressing the second research question—Are e-cigarette brands targeting sub-populations (eg, women, teenagers) by taking advantage of the demographic differences of different social networking sites?—we found no data suggesting direct targeting of sub-populations based on social networking site. The results of the 1-case view found it was likely that some of the sites were being used in different ways, but not in a manner consistent with focusing on any specific demographics. We followed up by analyzing the data from each site during specific holidays that might show a favoring of announcements or advertisements for one site over another and combined this information with the trends in different demographics reported by Pew on each of the sites [20]. For example, we examined the content during Mother’s Day, Father’s Day, Cinco de Mayo, and Black History Month, but found that the companies either ignored the holiday or were consistent in their celebration notices across all sites.

In addressing the third research question—Are there identifiable instances of users and information crossing different social networking sites?—we found a small percentage of users that had identical screen names on multiple sites, with the highest percentage being from Instagram/Twitter (128). These numbers differed from Pew’s report that multi-site users tended to use Facebook with another platform [20], although the numbers are too small to provide real contradictory evidence, and our study used a conservative comparison of screen names and profiles. We found no direct results of users acting on content by Blu or V2 leading to posting the same content on another site. We also discovered that matching user activity across different platforms can be problematic, as different types of activity were collected based on what is available from each site: Facebook’s posts, comments, and likes; Twitter’s retweets, Instagram’s comments and likes; and Google+’s resharers and plusoners (Table 5). Our findings will greatly support future research in multi-social networking site studies, as we can build on how to find users, analyze content, and normalize different activities.

## Conclusion

The purpose of this study was to examine the marketing strategies of leading e-cigarette brands on multiple social networking sites. We found that the two brands, Blu and V2, utilize different methods in how they interact with their customers. While Blu tends to be more conversational, V2’s focus is to direct people to their website. We did not find any evidence that either brand targets vulnerable populations in their strategies. However, we are able to see that Blu harnesses the affordances of different sites to conduct different types of marketing to their customer base. Our results demonstrate that a content analysis of multiple social networking sites can serve to identify how companies or brands can vary their marketing strategies based on the available technologies. We plan on expanding the foundation of this methodology to further understand potential diffusion of information across multiple sites.

There are several limitations in this study. Primarily, we focused on only two e-cigarette brands. While their dominant online presence made them attractive candidates, we are also interested in following up with additional brands. Similarly, we only chose four social networking sites. Other social networking sites such as Pinterest, Tumblr, and LinkedIn were considered but were not included due to various limitations. We also did not interview any of the users in the study for specific reactions to or comments on the messages by the e-cigarette brands. Presenting the user side and experience would help provide additional context to the media messages. However, two of the authors are involved in a parallel project that, informed by some of the results of this pilot, will involve such interviews. Relatedly, we limited our content to publicly available data. While this affords easy access since all of the text or images posted can be viewed by anyone online, we do not know the impact on the larger online audience, which includes users that restrict access to their accounts. Therefore, the ability to generalize the results should be limited to public user accounts. Lastly, our content analysis focused on individual terms and we did not develop a synonym set of conceptually equivalent themes prior to the analysis. While this allows for a cleaner analysis, it risks over-representation of certain concepts.

We strongly believe cross-platform studies will be a vital area of research in the future. There are apps available that allow users to streamline their post activity by broadcasting content, whether text, image, or other media, to multiple social networking sites at once (eg, Everypost, HootSuite). New social networking sites are also continuing to be developed, especially as technology provides new opportunities in other hardware, such as mobile phones or wearable devices. A fast-paced arena makes single-platform studies very narrow and difficult to generalize, as more of the population continues using multiple social networking sites [20]. Additionally, public health campaigns have begun utilizing social networking sites as a platform for their causes, although not always with the desired outcomes [24]. As researchers, we must be able to adapt to the changing landscape of social media tools. In particular, public health researchers should be made aware what sites might be susceptible to potentially dangerous marketing strategies. Our research in multi-social networking site marketing should only be considered a first step in understanding this type of analysis. New tools in social media will require constant vigilance by those in public health to not only understand new arenas, but also to quickly develop strategies for prevention and intervention. Researchers need to understand what tools are available to establish counter-measures, whether against demographic targeting or misinformation. Public health campaigns should be carried out across the social networking sites that will best reach influential users able to spread and diffuse messages across many sites. Researchers must know what affordances need to be focused on and how users are affected, rather than blindly choose their platforms and audience.
